# Domänenübergreifende diagnostische Genauigkeit eines DL‐CNN: Dermatoskopie versus klinische Nahaufnahmen

**DOI:** 10.1111/ddg.15900_g

**Published:** 2026-04-08

**Authors:** Anastasia Sophie Vollmer, Julia Katharina Winkler, Katharina Susanne Kommoss, Wilhelm Stolz, Albert Rosenberger, Alexander Enk, Holger Andreas Haenssle

**Affiliations:** ^1^ Universitäts‐Hautklinik Universitätsklinikum Heidelberg; ^2^ Hautklinik Klinik Thalkirchner Straße, München; ^3^ Abteilung für Genetische Epidemiologie Universitätsklinikum Georg‐August‐Universität Göttingen

**Keywords:** DL‐CNN, Dermatoskopie, klinische Bilder, Robustheit, Teledermatologie, Clinical images, dermoscopy, DL‐CNN, robustness, teledermatology

## Abstract

**Hintergrund und Zielsetzung:**

*Deep‐learning*‐basierte *Convolutional Neural Networks* (DL‐CNN) erzielen innerhalb der Domäne der dermatoskopischen Bildgebung eine hohe diagnostische Genauigkeit. In der klinischen Praxis stehen dermatoskopische Geräte jedoch nicht immer zur Verfügung, sodass häufig klinische Nahaufnahmen angefertigt werden. Diese Studie untersucht die domänenübergreifende diagnostische Genauigkeit eines mit dermatoskopischen Bildern trainierten DL‐CNN bei der Anwendung auf klinische Nahaufnahmen.

**Methoden:**

In dieser Querschnittsstudie wurde das DL‐CNN Moleanalyzer pro (trainiert mit 129 487 dermatoskopischen Bildern) an 350 Hautläsionen getestet, die sowohl klinisch als auch dermatoskopisch aufgenommen wurden. Referenzdiagnosen basierten auf der Histopathologie (89,4%) oder einem Expertenkonsens mit zweijährigem Follow‐up (10,6%). Primäre Endpunkte waren Sensitivität, Spezifität und die Fläche unter der ROC‐Kurve (ROC‐AUC).

**Ergebnisse:**

Für dermatoskopische Bilder erreichte das DL‐CNN eine Sensitivität von 88,2% (95%‐KI: 82,9%–92,0%), eine Spezifität von 69,0% (61,4%–75,8%) und eine ROC‐AUC von 0,866 (0,860–0,873). Bei den klinischen Nahaufnahmen derselben Läsionen sank die Sensitivität signifikant auf 60,5% (53,5%–67,1%, *p* < 0,001), während die Spezifität auf 79,4% (72,3%–85,0%, p = 0,027) anstieg. Die ROC‐AUC für Nahaufnahmen betrug 0,780 (0,772–0,790, p = 0,003).

**Schlussfolgerungen:**

Die domänenfremde Anwendung auf klinische Nahaufnahmen führte zu einer reduzierten Sensitivität bei gleichzeitig erhöhter Spezifität. Dies unterstreicht die Notwendigkeit, die domänenübergreifende Anpassungsfähigkeit von DL‐CNN‐Modellen zu verbessern. Ein *Fine‐Tuning* der DL‐CNN auf klinische Nahaufnahmen könnte die diagnostische Genauigkeit im klinischen Alltag erhöhen.

## EINLEITUNG

Kutane Malignome zählen zu den häufigsten Neoplasien in westlichen Bevölkerungen und stellen eine erhebliche Belastung für die individuelle Gesundheit sowie für die Gesundheitssysteme dar.^1^ Aufgrund des demografischen Wandels hin zu einer alternden Bevölkerung ist zu erwarten, dass sich diese Problematik künftig weiter verschärfen wird.^1^, ^2^

Die Dermatoskopie verbessert insbesondere bei pigmentierten Läsionen die diagnostische Genauigkeit in der Hautkrebsvorsorge.^3^, ^4^, ^5^ Ihre Verfügbarkeit ist jedoch in der primärärztlichen Versorgung, der Teledermatologie sowie in mobilen Gesundheitsanwendungen zur Selbstuntersuchung nach wie vor eingeschränkt.^6^ Klinische Nahaufnahmen, die einfacher anzufertigen sind, stellen in diesen Kontexten eine praxisnahe Alternative dar.

Gleichzeitig gewinnen *Deep‐learning*‐basierte *Convolutional Neural Networks* (DL‐CNN) zunehmend an Bedeutung als unterstützende Tools zur Bilderkennung in der Hautkrebsdiagnostik in Form einer *human‐with‐machine collaboration*.^7^, ^8^ Obwohl DL‐CNN insbesondere für melanozytäre Läsionen eine hohe diagnostische Genauigkeit gezeigt haben, vor allem wenn sie auf Dermatoskopiebildern trainiert und getestet wurden,^7^, ^8^, ^9^, ^10^, ^11^, ^12^, ^13^ lässt sich diese diagnostische Leistung bislang nur eingeschränkt aus kontrollierten Forschungsszenarien in den klinischen Alltag übertragen.^6^ Im Gegensatz zum menschlichen visuellen Kortex mangelt es bildverarbeitenden KI‐Architekturen häufig an Robustheit, insbesondere bei leichten Variationen in der Bildmorphologie oder beim Hauttyp.^14^, ^15^ Konvolutionale KI‐Architekturen gelten dabei als besonders anfällig gegenüber Bildstörungen wie Translationen oder Skalierungen.^14^, ^16^ Auch wenn eine erweiterte Datenaugmentation während des Trainings diese Schwächen teilweise kompensieren kann, bleibt ihre Effektivität begrenzt, sobald Testbilder signifikant von der Trainingsgrundlage abweichen.^16^, ^17^, ^18^ Die tatsächliche domänenübergreifende Robustheit marktzugelassener DL‐CNN, die in der Regel anhand von dermatoskopischem Bildmaterial trainiert wurden, ist bislang nur unzureichend untersucht. Zudem basieren viele dieser Modelle möglicherweise noch nicht auf den neuesten hybriden KI‐Architekturen, die derzeit in der Forschung erprobt werden.^19^, ^20^, ^21^ Diese Studie vergleicht systematisiert die dichotome Klassifikationsleistung eines marktzugelassenen DL‐CNN anhand gepaarter klinischer Nahaufnahmen und dermatoskopischer Bilder derselben Läsion. Dadurch lassen sich modalitätsabhängige Unterschiede in Sensitivität und Spezifität analysieren und praxisrelevante Aussagen zur klinischen Anwendbarkeit treffen. Die Ergebnisse liefern Impulse zur Verbesserung der Robustheit von DL‐CNN, insbesondere für Anwendungsbereiche wie Ganzkörperfotografie, Teledermatologie, Smartphone‐Apps, webbasierte Diagnostik und das Screening in der Primärversorgung.^6^, ^22^, ^23^, ^24^

## MATERIAL UND METHODIK

Diese Querschnittsstudie wurde von der zuständigen Ethikkommission genehmigt (S‐629/2017) und im Einklang mit der *Deklaration von Helsinki* durchgeführt. Getestet wurde ein binäres DL‐CNN (Moleanalyzer pro, FotoFinder Systems Inc.), basierend auf der Inception_v4‐Architektur von Google. Das DL‐CNN wurde mit 129 487 dermatoskopischen Bildern trainiert (100 021 benigne, 29 466 maligne Läsionen). Weitere Informationen finden sich im Online‐Supplement (M1). Das Modell generierte für jede Läsion einen Malignitätswert zwischen 0 und 1 (Grenzwert für Malignität ab > 0,5). Im Rahmen des DL‐CNN‐Preprocessings wurden alle Bilder auf eine einheitliche Größe von 299 × 299 Pixeln skaliert. Bilder wurden zusätzlich zugeschnitten, um potenzielle Störfaktoren wie Hautmarkierungen^25^ und ein dunkles Randartefakt^26^ zu minimieren (Abbildung S1 im Online‐Supplement).

### Test‐Set

**ABBILDUNG 1:**
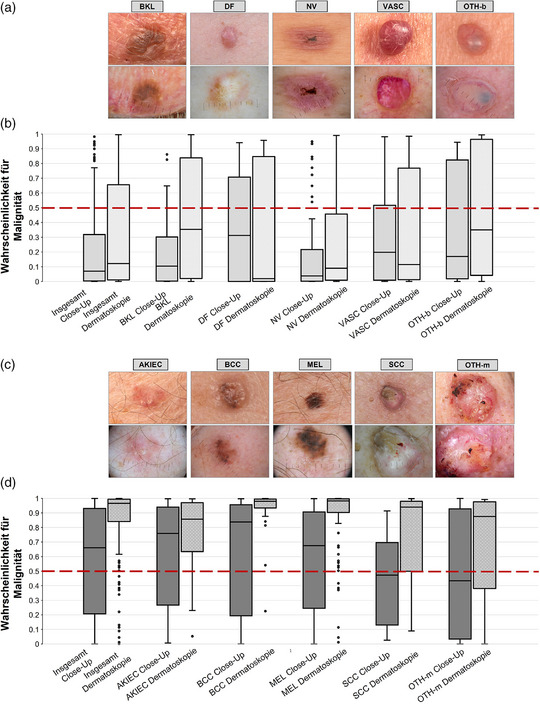
Repräsentative klinische Nahaufnahmen (Close‐up) mit darunter angeordneten korrespondierenden dermatoskopischen Bildern von benignen (a) und malignen (c) Entitäten. (a) Benigne Entitäten, von links nach rechts: Lentigo solaris und seborrhoische Keratose (BKL), Beispiel seborrhoische Keratose; Dermatofibrom (DF), Beispiel Dermatofibrom; Nävi (NV), Beispiel Recurrence‐Nävus; benigne vaskuläre Tumoren wie Angiom und pyogenes Granulom (VASC), Beispiel Hämangiom; sowie weitere benigne Läsionen wie Beispiel Spiradenom (OTH‐b). (c) Prämaligne oder maligne Entitäten, von links nach rechts: Aktinische Keratose/Morbus Bowen (AKIEC), Beispiel aktinische Keratose; Basalzellkarzinom (BCC), Beispiel pigmentiertes BCC; Melanom (MEL), Beispiel Melanoma in situ; spinozelluläres Karzinom einschließlich Morbus Bowen (SCC), Beispiel SCC; sowie weitere maligne Läsionen wie Beispiel Melanommetastasen (OTH‐m), Beispiel kutane Brustkrebsmetastase. (b, d) Vom DL‐CNN generierte Malignitätswerte (Skala 0 bis 1, Malignitätsverdacht ab > 0,5) für benigne (b) und maligne (d) Entitäten als Boxplots. Die linken Boxplots zeigen die Malignitätswerte für klinische Nahaufnahmen, die rechten für dermatoskopische Bilder. Höhere Werte (näher an 1) deuten auf eine höhere Wahrscheinlichkeit für Malignität hin; der Schwellenwert für Malignität liegt bei > 0,5 (rote gestrichelte Linie). Die Boxen repräsentieren den Interquartilsabstand (25.–75. Perzentil), die Medianlinie ist innerhalb der Box dargestellt. Die Whisker zeigen die Minimal‐ und Maximalwerte innerhalb der Verteilung an.

Eine Stichprobe von 350 Hautläsionen (195 maligne, 155 benigne) wurde aus der Bilddatenbank der Universitäts‐Hautklinik Heidelberg zusammengestellt. Eingeschlossen wurden verschiedene Entitäten, die im Rahmen der regulären klinischen Versorgung zwischen 2014 und 2024 aufgenommen wurden. Es gab keine Überschneidung zwischen dem Trainings‐ und Testdatensatz. Jede Läsion wurde sowohl klinisch (Nahaufnahme) als auch dermatoskopisch aufgenommen. Die Bildakquisition erfolgte mit unterschiedlichen Einstellungen der Kameras und der Dermatoskope. Dermatokopiebilder wurden mit polarisiertem Licht aufgenommen. Von den 350 klinischen Aufnahmen wurden 39 mit einem Smartphone aufgenommen. Insgesamt wurden 321 Diagnosen (91,7%) histopathologisch gesichert, während 29 Diagnosen (8,3%) nicht‐exzidierter Läsionen durch Expertenkonsens (formale Übereinstimmung unter drei erfahrenen Fachärzten) in Kombination mit einem unauffälligen Verlauf über mindestens 2 Jahre gestellt wurden. Letzteres traf ausschließlich auf benigne Läsionen zu. Der Datensatz umfasste pigmentierte und nichtpigmentierte Läsionen verschiedener Körperregionen (Tabelle 1). Benigne Läsionen beinhalteten keratinozytäre Läsionen wie Lentigo solaris und seborrhoische Keratose (BKL), Dermatofibrome (DF), Nävi (NV), benigne vaskuläre Entitäten wie Angiome und Granuloma pyogenicum (VASC) sowie weitere benigne Entitäten wie Spiradenome (OTH‐b) (Tabelle 1, Abbildung 1a). Die malignen Läsionen umfassten aktinische Keratose/Morbus Bowen (AKIEC), Basalzellkarzinome (BCC), Melanome (MEL), spinozelluläre Karzinome einschließlich Bowen‐Karzinome (SCC) sowie weitere maligne Entitäten, zum Beispiel Melanommetastasen (OTH‐m) (Tabelle 1, Abbildung 1c).

**TABELLE 1:** Charakteristika der Hautläsionen im Testdatensatz (n = 350).

	Benigne Entitäten (n = 155)		Maligne Entitäten (n = 195)	

	*n*	*%*	*n*	*%*
***Lokalisation***				
Kopfhaut	11	7.1	10	5.1
Gesicht/Lippe	34	21.9	61	31.3
Genital	‐	‐	1	0.5
Rumpf	64	41.3	87	44.6
Extremitäten	35	22.6	28	14.4
Akral	11	7.1	8	4.1
***Referenzstandard***				
*Histopathologie*	127	81.9	194	99.5
*Expertenkonsens und*	28	18.1	1^*^	0.5
*unauffälliges Follow‐up (> 2 Jahre)*				
***AKIEC***			**15**	**7.7**
Aktinische Keratose			8	4.1
Morbus Bowen			7	3.6
*Histopathologie*			14/15	93.3
***BCC***			**35**	**18**
Fibroepitheliom vom Pinkus‐Typ			1	0.5
BCC			32	16.4
Kollision BCC und Hämangiom			1	0.5
Kollision BCC und Nävus			1	0.5
*Histopathologie*			35/35	100
***MEL***			**106**	**54.4**
In situ Melanome				
Melanoma in situ			8	4.1
Lentigo maligna			10	5.1
Invasive Melanome				
Lentigo maligna Melanom			7	3.6
amelanotisch			2	1
nodulär			11	5.6
akrolentiginös			4	2.1
spitzoid			6	3.1
desmoplastisch			1	0.5
superfiziell spreitend			25	12.8
im histologischen Bericht nicht spezifiziert			32	16.4
*Histopathologie*			106/106	100
***SCC***			**6**	**3.1**
SCC			5	2.6
Bowen‐Karzinom			1	0.5
*Histopathologie*			6/6	100
***OTH‐m***			**33**	**16.9**
Metastase				
Primarius: Bronchialkarzinom			3	1.5
Primarius: Mammakarzinom			1	0.5
Primarius: Melanom			19	9.7
Atypisches Fibroxanthom			2	1
PDS			3	1.5
Mycosis fungoides			1	0.5
Merkelzellkarzinom			3	1.5
Kaposi‐Sarkom			1	0.5
*Histopathologie*			33/33	100
***BKL***	**27**	**17.4**		
Seborrhoische Keratose	21	13.6		
Solare Lentigo	6	3.9		
*Histopathologie*	25/27	92.6		
***DF***	**6**	**3.9**		
*Histopathologie*	5/6	83.3		
***NV***	77	49.7		
Blauer Nävus	11	7.1		
Spitz Nävus	5	3.2		
Dysplastischer Nävus	12	7.7		
Rezidiv Nävus	3	1.9		
Plantarer Nävus	1	0.7		
Becker Nävus	1	0.7		
Compound Nävus	15	9.7		
Papillomatöser Nävus	4	2.6		
Kombinierter Nävus	6	3.9		
Mechanisch irritierter Nävus	6	3.9		
Kongenitaler Nävus	13	8.4		
*Histopathologie*	56/77	72.7		
***VASC***	27	17.4		
Hämangiom	18	11.6		
Angiofibrom	1	0.7		
Granuloma Pyogenicum	5	3.2		
Angiokeratoma circumscriptum	1	0.7		
Angiokeratom	2	1.3		
*Histopathologie*	25/27	92.6		
***OTH‐b***	18	11.6		
Spiradenom	2	1.3		
Zylindrom	1	0.7		
Poroides Hidradenom	1	0.7		
Kutanes Pseudolymphom	1	0.7		
Klarzellakanthom	2	1.3		
Plantares Kollagenom (Proteus‐Syndrom)	1	0.7		
Condyloma acuminatum	1	0.7		
Juveniles Xanthogranulom	1	0.7		
Nävus sebaceus	1	0.7		
Talgdrüsenadenom	4	2.6		
Trichofollikulom	1	0.7		
Trichoblastom	1	0.7		
Verruca vulgaris	1	0.7		
Histopathologie	16/18	88.9		

* Klinisch diagnostizierte AK.

*Abk*.: AKIEC, aktinische Keratose/Morbus Bowen; BCC, Basalzellkarzinom; BKL, benigne Keratose; DF, Dermatofibrom; DL‐CNN, Deep‐Learning‐Convolutional‐Neural‐Network; MEL, Melanom; NV, Nävus; OTH‐b, sonstige benigne Läsion; OTH‐m, sonstige maligne Läsion; PDS, pleomorphes dermales Sarkom; ROC, receiver operating characteristic; SCC, spinozelluläres Karzinom; VASC, vaskuläre Läsion einschließlich Angiom und pyogenem Granulom

### Statistische Auswertung

Primäre Endpunkte umfassten Sensitivität, Spezifität und die Fläche unter der ROC‐Kurve (ROC‐AUC) für die binäre (benigne versus maligne) diagnostische Genauigkeit in der Klassifikation von Nahaufnahmen versus dermatoskopischen Bildern. Deskriptive Statistiken wurden als Häufigkeiten, Mittelwerte und Standardabweichungen angegeben. Zum paarweisen Vergleich von Sensitivitäten und Spezifitäten wurden zweiseitige McNemar‐Tests durchgeführt.^27^ Die ROC‐AUCs klinischer und dermatoskopischer Bilder wurden statistisch miteinander verglichen.^28^ Ein p‐Wert <0,05 wurde als statistisch signifikant gewertet. Die Analysen erfolgten mit SPSS Version 25 (IBM, SPSS; Chicago, IL, USA).

## ERGEBNISSE

### Charakteristika der Patienten und untersuchten Läsionen

Das mediane Alter der Patienten betrug 64,0 ± 21,9 Jahre (Bereich: 1–94 Jahre); 60,3% waren männlich. Die benignen Läsionen (n = 155) umfassten folgende diagnostische Kategorien: BKL (n = 27; 17,4%), DF (n = 6; 3,9%), NV (n = 77; 49,7%), VASC (n = 27; 17,4%) und OTH‐b (n = 18; 11,6%) (Tabelle 1). Die malignen Läsionen (n = 195) umfassten: AKIEC (n = 15; 7,7%), BCC (n = 35; 18,0%), MEL (n = 106; 54,4%), SCC (n = 6; 3,1%) sowie OTH‐m (n = 33; 16,9%) (Tabelle 1). Die meisten benignen und malignen Läsionen waren am Stamm lokalisiert (41,3% beziehungsweise 44,6%).

### Diagnostische Genauigkeit des DL‐CNN bei klinischen Nahaufnahmen versus dermatoskopischen Bildern

**ABBILDUNG 2:**
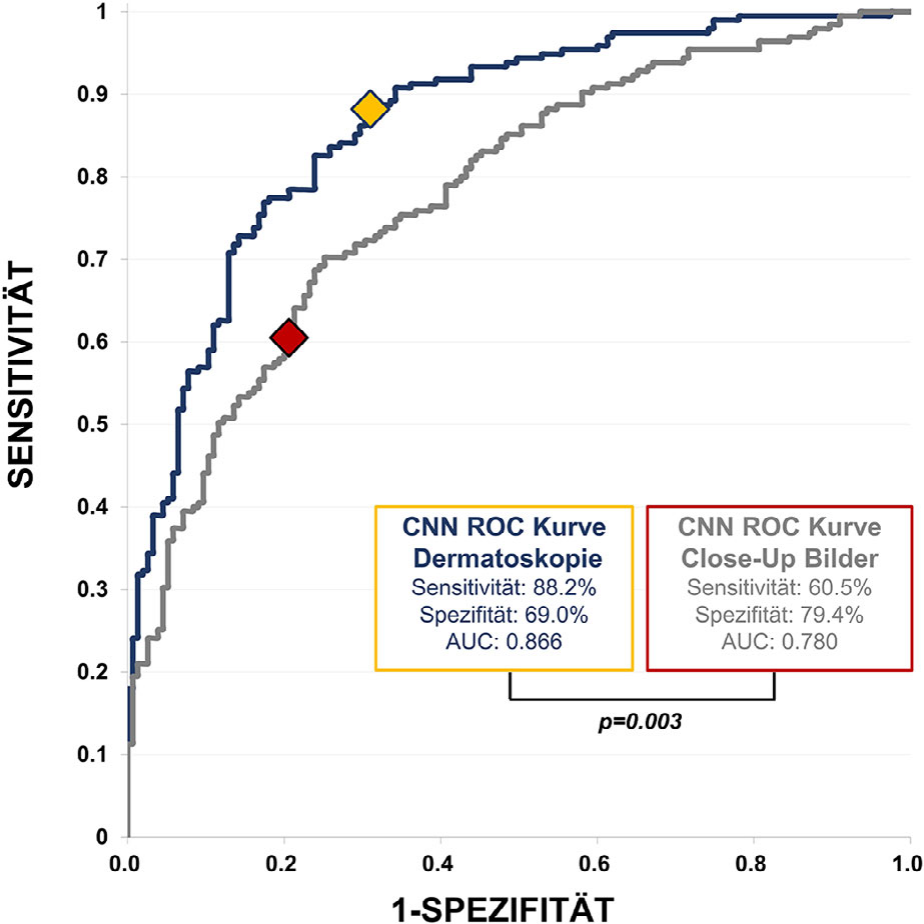
Receiver‐operating‐characteristic (ROC)‐Kurve des DL‐CNN für die binäre Klassifikation benigner vs. maligner Entitäten: Dermatoskopiebilder (blaue Kurve) vs. klinische Nahaufnahmen (graue Kurve). Der jeweilige Arbeitspunkt des DL‐CNN ist für die Dermatoskopie gelb und für die klinischen Bilder rot markiert. Die statistische Analyse ergab eine signifikant höhere diagnostische Genauigkeit für die ROC‐Kurve der Dermatoskopiebilder im Vergleich zu den klinischen Nahaufnahmen (p = 0,003).

Abbildung 1 zeigt Boxplots der vom DL‐CNN vergebenen Malignitätswerte für benigne (Abbildung 1b) und maligne Läsionen (Abbildung 1d), aufgeschlüsselt nach prädefinierten Diagnosegruppen. Insgesamt waren die Malignitätswerte für Nahaufnahmen bei benignen als auch bei malignen Läsionen deutlich niedriger als für dermatoskopische Bilder (linke Boxplots in Abbildung 1b, d), mit Ausnahme von DF und VASC bei benignen beziehungsweise BCC bei malignen Läsionen. Bei der Analyse dermatoskopischer Bilder erwies das DL‐CNN eine Sensitivität von 88,2% (95%‐Konfidenzintervall (KI): 82,9%–92,0%), eine Spezifität von 69,0% (61,4%–75,8%) und eine ROC‐AUC von 0,866 (0,860–0,873). Im Vergleich dazu sank die Sensitivität bei klinischen Nahaufnahmen signifikant um circa 30% auf 60,5% (53,5%–67,1%; *p* < 0,001), während die Spezifität um 10% auf 79,4% (72,3%–85,0%; p = 0,027) anstieg. Dies führte insgesamt zu einer signifikant reduzierten ROC‐AUC von 0,780 (0,772–0,790; p = 0,003) (Abbildung 2). Sensitivität und Spezifität für jede Diagnoseklasse sind in der Tabelle S1 (siehe Online‐Supplement) dargestellt. Zur weiteren Charakterisierung der diagnostischen Leistungsfähigkeit des DL‐CNN wurden Sensitivität und Spezifität zusätzlich für pigmentierte Läsionen nach Bildmodalität ausgewertet. Die Sensitivität war bei dermatoskopischen Bildern höher (92,5% (85,9%–96,2%)) als bei Nahaufnahmen (65,6%(54,1%–72,1%)), während die Spezifität bei Nahaufnahmen geringfügig höher ausfiel (84,8% (75,3%–91,1%) gegenüber 78,5% (68,2%–86,1%)). Zur Untersuchung der Auswirkungen einer Schwellenwertanpassung wurde die Leistung des DL‐CNN bei klinischen Bildern unter Verwendung des vordefinierten Malignitäts‐Schwellenwerts (0,5) mit dem optimierten Schwellenwert‐Index nach Youden (0,310) verglichen. Die Sensitivität stieg signifikant von 60,5% auf 69,7% (63,0%–75,8%; *p* < 0,001), während die Spezifität von 79,4% auf 74,8% (67,5%–81,0%; p = 0,008) sank. Die Ergebnisse deuten darauf hin, dass eine Schwellenwertoptimierung zu einer ausgewogeneren dichotomen Klassifikationsleistung führt. Dabei geht ein moderater Verlust an Spezifität mit einem klinisch relevanten Gewinn an Sensitivität einher, der potenziell die Zahl falsch‐negativer Diagnosen in der klinischen Praxis reduzieren kann. Im Vergleich zu dermatoskopischen Bildern blieb die Sensitivität trotz Schwellenwertanpassung signifikant niedriger (69,7% vs. 88,2%; *p* < 0,001), während kein signifikanter Unterschied in der Spezifität bestand (74,8% vs. 69,0%; p = 0,19).

### Analyse fehlklassifizierter Hautläsionen: Nahaufnahme versus Dermatoskopie

**ABBILDUNG 3:**
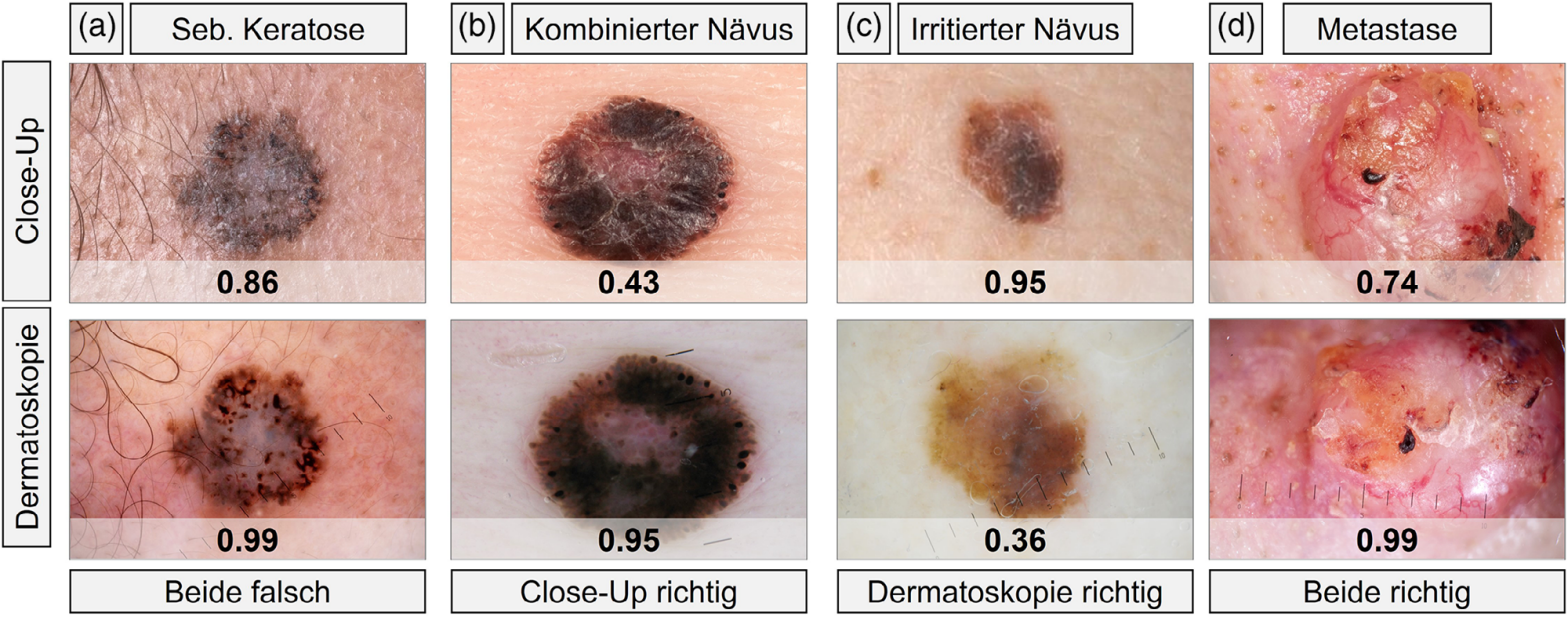
Repräsentative Läsionen zur Darstellung übereinstimmender und abweichender DL‐CNN‐Ergebnisse in klinischen Close‐up und dermatoskopischen Bildern. Jede Zeile zeigt die zugehörigen klinischen und dermatoskopischen Bilder derselben Läsion. (a) Fehlklassifikation in beiden Modalitäten (Beispiel seborrhoische Keratose). (b) Korrekte Klassifikation nur im klinischen Bild (Beispiel kombinierter Nävus mit compound‐ und Spitz‐Anteilen). (c) Korrekte Klassifikation nur im dermatoskopischen Bild (Beispiel mechanisch irritierter Nävus). (d) Korrekte Klassifikation in beiden Modalitäten (Beispiel kutane Metastase eines Mammakarzinoms).

Bei seltenen Entitäten, die im Trainingsdatensatz teils unzureichend repräsentiert waren, zeigte das DL‐CNN eine reduzierte Klassifikationsleistungen. Eine differenzierte Darstellung der Ergebnisse nach Diagnoseklasse (zum Beispiel Nävi, Dermatofibrome) findet sich in Tabelle 2. Die Anzahl korrekt klassifizierter Läsionen war bei dermatoskopischen Bildern höher (279/350; 79,7%) als bei Nahaufnahmen (241/350; 68,9%). In 203 Fällen wurde die Läsion in beiden Modalitäten korrekt klassifiziert, in 33 Fällen in beiden falsch, in 76 Fällen nur auf dem dermatoskopischen Bild korrekt und in 38 Fällen nur auf dem klinischen Bild (Abbildung 3). Bei dermatoskopischen Bildern trat eine höhere Rate falsch‐positiver Klassifikationen auf (48 von 155; 31,0%) im Vergleich zu Nahaufnahmen (32 von 155; 20,7%). Insbesondere wurden fast die Hälfte der VASC (13 von 27; 48,2%) und über ein Drittel der BKL (10 von 32; 37,1%) fälschlich als maligne eingestuft (Tabelle 2). Bei Nahaufnahmen wurden dagegen mehr falsch‐negative Klassifikationen beobachtet (77 von 195; 39,5%) als in der Dermatoskopie (23 von 195; 11,8%). Besonders häufig betroffen waren SCC (3 von 6; 50,0%) und OTH‐m Läsionen (18 von 33; 54,6%). Fehlklassifizierte OTH‐m‐Läsionen umfassten: Melanommetastasen (10 von 18; 55,6%), kutane Metastasen eines Bronchialkarzinoms (2 von 18; 11,1%), Merkelzellkarzinome (2 von 18; 11.1%), pleomorph dermale Sarkome (2 von 18; 11,1%), atypische Fibroxanthome (1 von 18; 5,6%) sowie Kaposi‐Sarkome (1 von 18; 5,6%). Insgesamt war die diagnostische Kategorie der Melanommetastasen, innerhalb der OTH‐m‐Kategorie, sowohl in klinischen Nahaufnahmen als auch in der Dermatoskopie die am häufigsten fehlklassifizierte. Bei der Analyse melanozytärer Läsionen zeigte sich, dass das CNN deutlich weniger Melanome in der Dermatoskopie (8 von 106; 7,6%) falsch klassifizierte als in klinischen Nahaufnahmen (38 von 106; 35,9%). Umgekehrt wurden Nävi in Nahaufnahmen seltener fälschlich als maligne eingestuft (10 von 77; 13,0%) als in der Dermatoskopie (17 von 77; 22,1%). Wurden Nahaufnahmen, die mittels Smartphone aufgenommen wurden, ausgeschlossen (39 von 350; 11,1%), sanken die Sensitivität und Spezifität des DL‐CNN bei Spiegelreflexkamera‐Aufnahmen leicht auf 59,9% (52,4%–66,9%) beziehungsweise 78,4% (70,9%–84,4%). Betrachtete man hingegen ausschließlich Nahaufnahmen, die mit einem Smartphone aufgenommen wurden, so ergab sich eine Sensitivität von 65,2% (44,9%–81,2%) und eine Spezifität von 100% (78,5%–100,0%).

**TABELLE 2:** Fehlklassifizierte Hautläsionen.

Fehlklassifizierte Hautläsionen		Close‐up		Dermatoskopie	
		*n/n*	*(%)*	*n/n*	*(%)*
*Falsch‐negative Klassifikationen*	*Falsch‐negativ‐Rate*	*77/195*	*(39.5)*	*23/195*	*(11.8)*
***AKIEC***		5/15	33.3	3/15	20
Aktinische Keratose		2/5	40.0	2/3	66.7
Morbus Bowen		3/5	60.0	1/3	33.3
***BCC***		13/35	37.1	1/35	2.9
Fibroepitheliom vom Pinkus‐Typ		1/13	0.1	–	–
BCC		12/13	92.3	1/35	2.9
***MEL***		38/106	35.9	8/106	7.5
Lentigo maligna		5/38	13.2	1/8	12.5
Melanoma in situ		3/38	7.9	2/8	25
Akrolentiginöses Melanom		1/38	2.6	2/8	25
Amelanotisches Melanom		1/38	2.6	–	–
Lentigo maligna Melanom		3/38	7.9	1/8	12.5
Noduläres Melanom		3/38	7.9	–	–
Spitzoides Melanom		2/38	5.3	1/8	12.5
Superfiziell spreitendes Melanom		11/38	28.9	–	–
Melanom nicht spezifiziert		9/38	23.7	1/8	12.5
***SCC***		3/6	50	1/6	16.7
Keratoakanthom		1/3	33.3	1/1	100
SCC		1/3	33.3	–	–
Bowen‐Karzinom		1/3	33.3	–	–
***OTH‐m***		18/33	54.6	10/33	30.3
AFX		1/18	5.6	–	–
PDS		2/18	11.1	–	–
Metastase eines Bronchialkarzinoms		2/18	11.1	–	–
Melanommetastase		10/18	55.6	10/10	100
Merkelzellkarzinom		2/18	11.1	–	–
Kaposi‐Sarkom		1/18	5.6	–	–

*Falsch‐positive Klassifikationen*	*Falsch‐positiv‐Rate*	32/155	(20.7)	48/155	(30.1)
***BKL***		5/32	15.6	10/32	31.3
Solare Lentigo		2/5	40	–	–
Seborrhoische Keratose		3/5	60	10/10	100
***DF***		3/6	50	2/6	33.3
***NV***		10/77	13	17/77	22.1
Dysplastischer Nävus		2/10	20	7/17	41.2
Kongenitaler Nävus		2/10	20	3/17	17.7
Blauer Nävus		2/10	20	1/17	5.9
Papillomatöser Nävus		1/10	10	3/17	17.7
Spitz‐Nävus		2/10	20	2/17	11.7
Rezidiv‐Nävus		1/10	10	1/17	5.9
***VASC***		7/27	25.9	13/27	48.1
Angiokeratom		1/7	14.3	1/13	7.7
Granuloma pyogenicum		1/7	14.3	1/13	7.7
Angiofibrom		1/7	14.3	1/13	7.7
Hämangiom		4/7	57.1	7/13	53.9
***OTH‐b***		7/18	38.9	6/18	33.3
Poroides Hidradenom		1/7	14.3	–	–
Trichoblastom		–	–	1/6	16.7
Zylindrom		1/7	14.3	1/6	16.7
Spiradenom		–	–	1/6	16.7
Talgdrüsenadenom		4/7	57.1	2/6	33.3
Kutanes Pseudolymphom		–	–	1/6	16.7
Klarzellakanthom		1/7	14.3	–	–

*Abk*.: AFX, atypisches Fibroxanthom; AKIEC, aktinische Keratose/Morbus Bowen; BCC, Basalzellkarzinom; BKL, benigne Keratose; DF, Dermatofibrom; MEL, Melanom; NV, Nävus; OTH‐b, sonstige benigne Läsion; OTH‐m, sonstige maligne Läsion; PDS, pleomorphes dermales Sarkom; SCC, spinozelluläres Karzinom; VASC, vaskuläre Läsion einschließlich Angiom und pyogenem Granulom

## DISKUSSION

Der Einsatz von DL‐CNN in der Hautkrebsdiagnostik wurde mit dem Ziel vorangetrieben, ein unterstützendes Instrument zur Verbesserung der diagnostischen Genauigkeit von Klinikern bereitzustellen.^8^, ^25^ Frühere Studien legen nahe, dass insbesondere weniger erfahrene oder nicht spezialisierte Anwender stärker von der Zusammenarbeit mit einem DL‐CNN profitieren.^8^ Die meisten Anwendungsfälle für DL‐CNN sind daher im hausärztlichen Bereich, in der Teledermatologie sowie bei der patientengestützten Selbstuntersuchung über Smartphone‐Apps zu erwarten.^6^ Der Zugang zu digitalen Dermatoskopen ist in diesen Settings jedoch häufig eingeschränkt, während klinische Nahaufnahmen leicht mit Smartphones oder Digitalkameras erstellt werden können. Darüber hinaus werden derzeit automatisierte Systeme zur Ganzkörperfotografie und 3D‐Hautbildgebung entwickelt, die KI‐gestützte Modelle zur Klassifikation von Hautläsionen in klinischen Bildern nutzen sollen.^29^

Unsere Studie liefert einen kritischen und systematisierten Vergleich der dichotomen Klassifikationsleistung eines marktzugelassenen DL‐CNN, das mit dermatoskopischen Bildern trainiert wurde, wenn es mit klinischen Nahaufnahmen derselben Läsionen konfrontiert wird. Die ROC‐AUC des DL‐CNN bei der Klassifikation von Dermatoskopiebildern war signifikant höher als bei klinischen Nahaufnahmen, was auf eine insgesamt bessere diagnostische Genauigkeit innerhalb der Domäne dermatoskopischer Bilder hinweist. Falsch‐negative Ergebnisse traten bei klinischen Nahaufnahmen deutlich häufiger auf (39,5%) als bei dermatoskopischen Bildern (11,8%), was angesichts der hohen Fehlklassifikationsrate insbesondere bei bestimmten malignen Entitäten wie Plattenepithelkarzinomen und anderen seltenen Hauttumoren (OTH‐m), einschließlich Melanommetastasen, problematisch ist. Dies unterstreicht die Grenzen einer ausschließlichen Nutzung klinischer Bilder im Hautkrebsscreening – insbesondere bei Hochrisikopatienten.

Interessanterweise erreichte das DL‐CNN bei professionell aufgenommenen Smartphone‐Bildern eine Spezifität von 100%. Trotz der begrenzten Stichprobengröße könnte dies auf eine systematische Verschiebung hin zu niedrigeren Malignitätswerten bei Bildern geringerer Qualität hindeuten, was potenziell zu noch mehr übersehenen Diagnosen und verzögerter Behandlung führen kann.^22^ Zwar war die Sensitivität bei Smartphone‐Bildern geringfügig höher als bei Spiegelreflexkamera‐Aufnahmen (65,2% vs. 59,9%), doch der markante Trade‐off mit der Spezifität unterstreicht zusätzlich das Risiko falsch‐negativer Befunde. Diese Ergebnisse zeigen, dass die diagnostische Genauigkeit auch unter professionellen Aufnahmebedingungen von der verwendeten Bildmodalität abhängen kann und betonen die Wichtigkeit standardisierter Bildqualität sowie gezielter Patientenschulung, insbesondere in der Teledermatologie.

Mehrere DL‐CNN, die auf dermatoskopischen Bildern trainiert wurden, konnten in früheren Studien eine hervorragende Klassifikationsleistung bei der Melanomdiagnostik sowie bei anderen Hauttumoren zeigen – teils sogar besser als Dermatologen.^9^, ^10^, ^11^, ^12^, ^25^ Im Gegensatz dazu existieren nur wenige Studien, die das Training von DL‐CNN mit klinischen Bildern zur Hautkrebsdiagnostik untersuchen. Im Jahr 2020 berichteten Birkenfeld et al. über eine diagnostische Genauigkeit von 75,9% für die Identifikation suspekter pigmentierter Läsionen in klinischen Aufnahmen, wobei sie allerdings ein mehrstufiges Modell einsetzten.^30^ Weitere Studien zum Einsatz von DL‐CNN auf klinische Nahaufnahmen zeigten inkonsistente Ergebnisse. Nasr‐Esfahani et al. zeigten 2016 eine Sensitivität von 81% und eine Spezifität von 80%,^31^ während Han et al. 2018 eine Sensitivität von 91% und eine Spezifität von 90% erreichten.^31^ Die Unterschiede zwischen den Studien sind am ehesten auf die Heterogenität der Datensätze und Methodologien zurückzuführen.

In einer aktuellen Studie von Brinker et al. wurde ein ausschließlich mit Dermatoskopiebildern trainiertes DL‐CNN mit der diagnostischen Leistung von 145 Dermatologen auf einem Testdatensatz mit 100 klinischen Bildern verglichen.^11^ Trotz des Domänenwechsels erreichte das DL‐CNN eine Sensitivität von 89,4% und eine Spezifität von 68,2%, was vergleichbar mit den Ergebnissen der Dermatologen war. Im Gegensatz zu Brinker et al. nutzten wir ein breiteres Spektrum an Diagnosen und führten einen direkten Vergleich klinischer und dermatoskopischer Bilder derselben Läsionen durch, um Genauigkeitsunterschiede besser zu erfassen. Eine weitere wichtige Studie von Tschandl et al.^33^ untersuchte ein kombiniertes CNN (cCNN), das mit dermatoskopischen und klinischen Bildern nicht‐pigmentierter Hautläsionen trainiert wurde.^33^ Auch hier zeigte sich eine vergleichbare Leistung zum Durchschnitt von 95 Klinikern. Unsere Ergebnisse zeigen, dass das DL‐CNN maligne Läsionen auf dermatoskopischen Bildern besser erkannte als auf klinischen Nahaufnahmen, während die diagnostische Leistung für benignen Läsionen in klinischen Bildern höher war.

Darüber hinaus offenbarte unser DL‐CNN eine niedrigere Genauigkeit bei seltenen Hauttumoren, insbesondere bei klinischen Nahaufnahmen, was auf eine „Blind‐Spot“‐Problematik durch Unterrepräsentation in den Trainingsdaten hinweisen könnte.^17^, ^18^ In einer systematischeren Herangehensweise verglichen Rios‐Duarte et al. drei DL‐CNN, die entweder mit klinischen Bildern, mit Dermatoskopiebildern oder mit gepaarten Bildern derselben Läsionen trainiert wurden.^34^ In einem einheitlichen Testsatz mit 119 gepaarten Fällen zeigte sich, dass das ausschließlich auf Dermatoskopie trainierte Modell die höchste ROC‐AUC von 0,869 erreichte, gefolgt vom kombinierten Modell (cCNN) (0,822). Beide Modelle mit Dermatoskopiebildern im Training schnitten deutlich besser ab als das nur auf klinischen Bildern trainierte Modell (ROC‐AUC 0,661; *p* < 0,001).^34^ Unsere Daten bestätigen diese Beobachtung: Melanome wurden seltener in Dermatoskopiebildern als in klinischen Nahaufnahmen fehlklassifiziert (7,6% vs. 35,9%), während benigne Nävi in klinischen Nahaufnahmen seltener fälschlich als maligne klassifiziert wurden (13,0% vs. 22,1% in der Dermatoskopie). Dies verdeutlicht: Während die Dermatoskopie für die Detektion von Melanomen unerlässlich bleibt, könnten klinische Nahaufnahmen eine konservativere Managementstrategie bei low‐risk Patienten unterstützen.

Die Gesamtheit dieser Daten legt nahe, dass ein robustes DL‐CNN‐Training auf detailreiche Bilddomänen wie dermatoskopische Bilddatensätze essenziell für eine hohe diagnostische Leistung ist. Solch detailreiche, erlernte „Repräsentationsmasken“ lassen sich dann auch eher erfolgreich auf detailärmere (Ziel‐)Bilddomänen übertragen. Umgekehrt funktioniert dieser Transfer demnach weniger gut: Das Training auf klinische Bilder mit anschließender Anwendung auf dermatoskopische Bilder bringt deutlich schlechtere Ergebnisse. Der beobachtete Anstieg korrekt klassifizierter benigner Läsionen in klinischen Bildern um etwa 10 % erscheint weiter untersuchenswert, etwa durch den Einsatz kombinierter KI‐Modelle („Ensembles“). Ebenso könnte der beobachtete Sensitivitätsverlust von 30% durch Transferlernen abgeschwächt werden, etwa durch gezieltes *Fine‐Tuning* des DL‐CNN auf klinische Nahaufnahmen. Unsere Analyse zur Schwellenwertoptimierung unterstreicht darüber hinaus die Bedeutung/Wichtigkeit der Modellkalibrierung in spezifischen klinischen Kontexten.^20^ Eine Anpassung des Malignitätsschwellenwerts ermöglichte ein ausgewogeneres Verhältnis von Sensitivität/Spezifität und ermöglichte einen klinisch relevanten Zugewinn an Sensitivität. Eine sorgfältige Feinjustierung könnte somit helfen, falsch‐negative Diagnosen zu reduzieren, insbesondere dann, wenn nur klinische Nahaufnahmen als Bildquelle zur Verfügung stehen.

Ein möglicher Limitationsfaktor unserer Studie besteht darin, dass kein direkter Vergleich mit Dermatologen vorgenommen wurde. Der Fokus lag jedoch darauf, die diagnostische Leistung des DL‐CNN für die dermatoskopischen Bilder als Referenzmaßstab heranzuziehen. Weitere Limitationen betreffen die vorwiegend helle Hautfarbe der rekrutierten Patientenbilder, die bei bestimmten Diagnosen mit variablen dermatoskopischen Mustern einhergehen kann,^35^ sowie durch die Verwendung unserer „Convenience“‐Stichprobe. Zudem wurde kein begleitendes Metadaten‐Set zur Klassifikation durch das DL‐CNN einbezogen (zum Beispiel Alter, Lokalisation, sequentielle Läsionsentwicklung). Diese Einschränkung wurde bewusst in Kauf genommen, da derzeitige DL‐CNN mit Martkzulassung zur Unterstützung im Hautkrebsscreening und nicht zur Therapieentscheidung vorgesehen sind.^8^ Die Auswahl einer hohen Anzahl maligner Läsionen wurde getroffen, um einen zuverlässigen histologischen Referenzstandard zu gewährleisten. Da Unterschiede zwischen klinischen und dermatoskopischen Bildern ein und derselben Läsion nicht nur auf unterschiedliche Aufnahmebedingungen, sondern auch auf Faktoren wie Skalierung, Rotation oder Bildartefakte zurückzuführen sein können, wurde ein standardisiertes Preprocessing durchgeführt, um diese Störvariablen zu minimieren.^14^, ^15^, ^16^, ^19^ Die Generalisierbarkeit unserer Ergebnisse könnte durch die ausschließliche Verwendung von Bildmaterial einer einzelnen Institution sowie eines spezifischen DL‐CNN‐Modells eingeschränkt sein. Dennoch ist die Aussagekraft der Studie hoch, da sie sich gezielt auf ein marktzugelassenes und im klinischen Alltag eingesetztes KI‐System konzentriert hat. Im Gegensatz dazu könnten neuere KI‐Architekturen, die in der Forschung eingesetzt werden, wie etwa jene von Minderer et al., eine höhere domänenübergreifende Robustheit aufweisen.^19^

Unsere Ergebnisse zeigen, dass ein auf dermatoskopische Bilder trainiertes DL‐CNN bei der Anwendung auf klinische Nahaufnahmen eine rund 30% niedrigere Sensitivität, jedoch eine um etwa 10% höhere Spezifität aufweist. Frühere Studien belegten, dass ein kombiniertes Training mit klinischen und dermatoskopischen Bildern im Vergleich zu einem rein dermatoskopiebasierten Training nur geringe Verbesserungen in der Sensitivität erzielt. Dies deutet darauf hin, dass die diagnostische Leistungsfähigkeit von DL‐CNN eingeschränkt ist, wenn ausschließlich klinische Nahaufnahmen als Bildquelle zur Verfügung stehen. Diese Einschränkung hat relevante Implikationen für den Einsatz KI‐gestützter Systeme in der Teledermatologie, in mobilen Applikationen und in der automatisierten Ganzkörperbildgebung. Ein Bilderkennungstool mit einer hohen Spezifität kann ein hilfreiches Triage‐Instrument zur Identifikation eindeutig benigner Läsionen in klinischen Nahaufnahmen sein. Für unklare oder potenziell maligne Hautveränderungen erscheint hingegen die höhere diagnostische Genauigkeit in detailreichem dermatoskopischem Bildmaterial unerlässlich. Der flächendeckende Zugang zu erschwinglichen digitalen Dermatoskopen, insbesondere in nicht spezialisierten Versorgungsbereichen, gewinnt damit an zentraler Bedeutung. Perspektivisch könnten DL‐CNN durch flexiblere und letztlich auch robustere Modellarchitekturen wie Vision Transformer (ViT) oder hybride CNN‐Transformer‐Modelle ersetzt werden, die für spezifische Aufgaben gezielt feinjustiert werden können.^20^, ^21^ Eine entscheidende Fragestellung bleibt dabei bislang unbeantwortet: Welcher Umfang an Fine‐Tuning ist erforderlich, um die Klassifikationsleistung für klinische Nahaufnahmen gezielt zu optimieren? Vergleichsstudien zwischen CNN, ViT und hybriden KI‐Architekturen könnten hierzu wertvolle Erkenntnisse liefern.

## FÖRDERUNG

Katharina Susanne Kommoss wird durch das Physician‐Scientist‐Programm der Medizinischen Fakultät der Universität Heidelberg gefördert. Die Förderung erfolgt unabhängig von der vorliegenden Studie.

## DANKSAGUNG

Open access Veröffentlichung ermöglicht und organisiert durch Projekt DEAL.

## INTERESSENKONFLIKT

H.A.H. erhielt Honorare und/oder Reisekostenerstattungen von Firmen, die Geräte zur Hautkrebsfrüherkennung entwickeln: Scibase AB, FotoFinder Systems GmbH, Heine Optotechnik GmbH, Magnosco GmbH. J.K.W. erhielt Honorare von FotoFinder Systems GmbH. W.S. erhielt Honorare von FotoFinder Systems GmbH und Heine Optotechnik GmbH. Die übrigen Autoren erklären, dass kein Interessenkonflikt im Zusammenhang mit dieser Studie besteht.

## Supporting information



Supplementary information

Supplementary information

Supplementary information

Supplementary information
